# Antioxidant Activity Evaluation of FlexirubinType Pigment from *Chryseobacterium artocarpi* CECT 8497 and Related Docking Study

**DOI:** 10.3390/molecules26040979

**Published:** 2021-02-12

**Authors:** Abeer Mogadem, Mohamed Ali Almamary, Naji Arafat Mahat, Khairunadwa Jemon, Wan Azlina Ahmad, Imran Ali

**Affiliations:** 1 Department of Chemistry, Faculty of Science, Universiti Teknologi Malaysia, Johor Bahru 81310 UTM, Johor, Malaysia; abeer.i.m@hotmail.com (A.M.); naji@kimia.fs.utm.my (N.A.M.); azlina@kimia.fs.utm.my (W.A.A.); 2 Department of Chemistry, Faculty of Science, Taibah University, Al-Madinah Al-Munawarah 42353, Saudi Arabia; mmamari@taibahu.edu.sa; 3 Department of Bioscience, Faculty of Science, Universiti Teknologi Malaysia, Johor Bahru 81310 UTM, Johor, Malaysia; khairun_nadwa@fbb.utm.my; 4 Department of Chemistry, Jamia Millia Islamia, A Central University, New Delhi 11025, India

**Keywords:** flexirubin, *Chryseobacterium artocarpi* CECT 8497, antioxidant activity, free radicals, molecular docking

## Abstract

The current research is focused on studying the biological efficacy of flexirubin, a pigment extracted from *Chryseobacterium artocarpi* CECT 8497.Different methods such as DPPH, H_2_O_2,_ NO^•^, O_2_^•−^, ^•^OH, lipid peroxidation inhibition by FTC and TBA, ferric reducing and ferrous chelating activity were carried out to evaluate the antioxidant activity of flexirubin. Molecular docking was also carried out, seeking the molecular interactions of flexirubin and a standard antioxidant compound with SOD enzyme to figure out the possible flexirubin activity mechanism. The new findings revealed that the highest level of flexirubin exhibited similar antioxidant activity as that of the standard compound according to the H_2_O_2_, ^•^OH, O_2_^•−^, FTC and TBA methods. On the other hand, flexirubin at the highest level has shown lower antioxidant activity than the positive control according to the DPPH and NO• and even much lower when measured by the FRAP method. Molecular docking showed that the interaction of flexirubin was in the binding cavity of the SOD enzyme and did not affect its metal-binding site. These results revealed that flexirubin has antioxidant properties and can be a useful therapeutic compound in preventing or treating free radical-related diseases.

## 1. Introduction

Small pharmaceuticals continue to be produced from natural sources via direct or semi-synthetic methods [1]. The animals, plants and microscopic organisms (algae, fungi, bacteria) are familiar sources of useful natural products with attractive biological and curative properties [2]. It has been reported that greater than 75% of the treatments used for infective illness are obtained from natural sources [3]. Among many benefits of microbes, the production of natural pigments has been acquiring popularity due to their commercial and medicinal values [4]. Microorganisms-derived pigments have been pointed up in diverse applications, such as food, cosmetics, textile and pharmaceutical industries [5]. Simultaneously, natural colorants have exhibited a wide spectrum of biological activities [6,7,8]. Previous researchers have indicated that the current knowledge is scarce in this context, and many natural resources with unknown pigmented compounds remain untapped [9].

**Figure 1:**
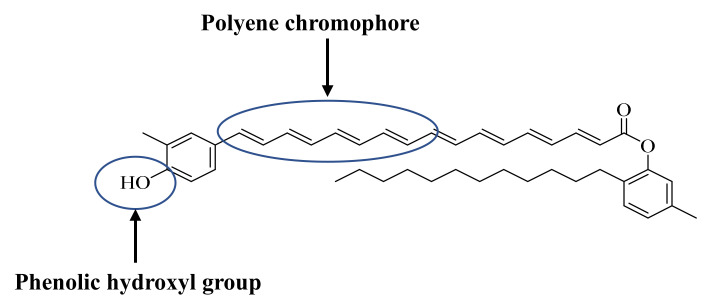
Flexirubin structure comprising of polyene chromophore and phenolic hydroxyl group proposed using ChemBioDraw Ultra 12.0.

Pigments with yellowish-orange color produced by bacteria include flexirubin, β-carotene astaxanthin and ankaflavin [10]. The studies on yellowish-orange pigments have recently been the interest of the scientific community. These bacterial pigments exhibit various biological activities with clinical relevance such as antioxidant, anticancer, antibacterial, antimalarial, antifungal, anti-inflammatory and anti-lipid activities [11]. Mostly, the mechanism of action of the mentioned activities remains unreported. Flexirubin type pigments produced by the bacterial species of *Chryseobacterium artocarpi* CECT8497 (from rhizosphere soil of *Artocarpus integer*) are yellow; the color can change reversibly to brown, red, or purple upon reacting with basic potassium hydroxide (KOH) solution. The color shift is due to the phenolic hydroxyl and polyene chromophore group in flexirubin structure (Figure 1). It is clear from Figure 1 that flexirubin has a long chain of carbon with alternative single and double bonds. The structure also has benzene rings, hydroxyl and ketonic groups. Although some activities of flexirubin that include antimicrobial and anticancer have been reported, specific investigations on its antioxidant and free radical scavenging are lacking [12,13].

Oxidative stress describes the imbalance between reactive oxygen species (ROS) production, such as hydroxyl radical, hydrogen peroxide, singlet oxygen, superoxide anion and nitric oxide [14] and the ability of an antioxidant defense mechanism to detoxify these reactive radicals [15].

This oxidative imbalance would cause severe damages, leading to modifications of biomolecules. These modifications include protein homeostasis disturbance, lipid peroxide formation and DNA mutation that reduce the cellular biological process capacity and elevate the risk of developing diseases such as cancers and neurodegenerative diseases [16]. Hence, the research about the interrelation between oxidative stress and related illness has become a popular research scope. The superoxide anion radical is an inevitable and cytotoxic byproduct of aerobic metabolism. Superoxide dismutases (SODs) metalloenzyme act as defense systems in all aerobes to avoid ROS’s extensive production, catalyze superoxide radicals’ disproportionation to hydrogen peroxide (H_2_O_2_) and molecular oxygen (O_2_)through oxidation and reduction alternation process [17]. SOD can also be used as an additional parameter for diagnosing oxidative stress-related disease [18]. Flexirubin isolated from *Chryseobacterium artocarpi* CECT 8497 has been classified as non-toxic in toxicity studies [19]; however, its antioxidant properties have not been fully evaluated. Hence, the present study aimed to evaluate the in vitro antioxidant activity of flexirubin and assess the possible interactions between flexirubin andthe active pocket of SOD enzyme using in silico molecular docking. The results of this study would provide, for the first time, the mode of action of flexirubin as an antioxidant compound besides its known role as a natural colorant.

## 2. Materials and Methods

### 2.1. Chemicals and Reagents

The chemicals and reagents used were of AR grade and obtained from the different companies. Nutrient broth (NB) and Silicon anti-foaming agent were purchased from Merck, Germany. Ascorbic acid, Trolox, diphenyl- 1-picrylhydrazyl radical (DPPH), hydrogen peroxide (H2O2), sulphanilamide, phosphoric acid, naphthyl ethylene diamine dihydrochloride (NEDD), sodium nitroprusside, nitro blue tetrazolium (NBT), phenazine methosulfate (PMS), nicotinamide adenine dinucleotide (NADH), ferrous ammonium sulphate, ethylenediamine tetra-acetic acid (EDTA), Thiobarbituric acid (TBA), tripyridyl triazine (TPTZ), ferrozine, dimethyl sulphoxide (DMSO), ammonium acetate, trichloroacetic acid (TCA), linoleic acid, ammonium thiocyanate, ferrous chloride, ferric chloride, hydrochloric acid (HCl), glacial acetic acid, sodium dihydrogen phosphate, disodium hydrogen phosphate, acetate buffer, phosphate buffer saline (PBS), ethanol, acetone, and methanol were purchased from Sigma-Aldrich Chemical Co. (St. Louis, MO, USA).

### 2.2. Instruments

The various instruments used are summarized in this section. 5L Bioreactor (BiotronLiflus GX 7 L, Gimpo-si, Gyeonggi-do, Korea), centrifuge (Allegra™ 25R Centrifuge-Beckman Coulter™, California, Massachusetts, USA), sonicator (QSonica, Newtown, USA), Rotary evaporator (STRIKE 300, Stereo Glass, San Martino in Campo-Perugia, Italy), UV–VIS spectrophotometer (Thermo Scientific™ GENESYS™ 10S), Autoclave (Hirayama HV-50, Japan).

### 2.3. Culture Conditions and Extraction of Flexirubin

A yellowish orange pigmented bacterial strain, *Chryseobacterium artocarpi* CECT 8497, was used in this study. It was previously isolated and characterized from rhizosphere soil samples associated with the woody tree Artocarpus integer from an orchard in Universiti Teknologi Malaysia (UTM), Malaysia [20]. The bacteria were maintained in nutrient agar and sub-cultured every month. The liquid culture was obtained by growing a loopful of *Chryseobacterium artocarpi* CECT 8497 culture in 50 mL nutrient broth (NB), incubated at 30 °C with the agitation rate of 200 rpm. After 24 h, the culture was cultivated by transferring 10% (*v/v*) inoculum to 450 mL NB and grown for 24 h (30 °C and 200 rpm). Finally, 500 mL (10%, *v/v*) of active culture was transferred into a 5 L bioreactor (Biotron, Incheon, Korea) containing 4500 NB under the following conditions: 30 °C, 200 rpm, aeration rate 2 L/min, initial pH 7.0 and with the addition of some Antifoam A (Sigma, Neustadt an der Weinstrasse, Germany) for 24 h. The culture broth was centrifuged at 10,000 rpm for 10 min (Allegra™ 25R Centrifuge-Beckman Coulter™, California, Massachusetts, USA), and the pellets were extracted using 5% acetone. The extraction was then sonicated twice (QSonica, Newtown, USA) for 20 s to break the cells to release the pigments. The extract was centrifuged at 8000 rpm for 10 min, and the supernatant containing the pigment was concentrated using a rotary evaporator at 50 °C dried for 3 days at 40 °C followed by separation using column chromatography using petroleum ether: benzene: acetone (10:40:5). The flexirubin extract currently used was of the same purity as confirmed and reported by a previously carried out work in the same laboratory used for this study [20].

### 2.4. In Vitro Antioxidant Assay

Firstly, 10 mM of flexirubin stock solution was prepared (by weighing 635 mg of flexirubin) in 100 mL of acetone as solvent. The stock solution was diluted to obtain different working solutions (0.05–10 mM) for further use in different assays. All analyses were done in triplicates.

#### 2.4.1. Scavenging Activity of DPPH (1,1-diphenyl-2-picrylhydrazyl) Radical

The power of flexirubin to scavenge DPPH free radicals was specified based on the procedures reported by previous researchers [21]. An aliquot of 0.1 mL of flexirubin (0.05–10 mM) was mixed with 2 mL of freshly prepared DPPH ethanolic solution (0.3 mM). After 30 min, the sample was incubated in the dark and the absorbance measured at 517 nm, with Trolox as the standard. The percentages of inhibition of the DPPH free radical were calculated using the equation below:

(1) $$\%\;\mathrm{Inhibition}\;=\;\frac{ABScontrol-ABStest}{ABScontrol}\times 100$$

where ABS control is the absorbance of the control solution (contains all the test reagents except the test compound) and ABStest is the absorbance of flexirubin samples or Trolox.

#### 2.4.2. Scavenging Activity of Hydrogen Peroxide (H_2_O_2_)

The free radical scavenging activity of flexirubin pigment was determined by hydrogen peroxide radical scavenging assay [22]. The hydrogen peroxide solution (43 mM) was prepared in phosphate buffer (0.1 M, pH 7.4). Flexirubin (0.1 mL) of different concentrations (0.05–10 mM) was rapidly mixed with 1 mL of hydrogen peroxide solution. The absorbance was measured at 230 nm using a UV spectrophotometer after 10min of incubation at 37 °C. H_2_O_2_ solution without the test sample was used as a control, while Trolox was used as the standard. The percentage scavenging of H_2_O_2_was calculated using the equation as in Section 2.4.1.

#### 2.4.3. Scavenging Activity of Nitric Oxide Radical (NO^−^)

In an aqueous solution at physiological pH, sodium nitroprusside automatically generates NO, which intermingles with oxygen to generate nitrite ions detected by the Griess reagent (1% sulphanilamide, 2% phosphoric acid and 0.1% naphthyl ethylene diamine dihydrochloride) [23]. The scavenging of free radicals would result in the reduced production of NO. In this assay, 3 mL of sodium nitroprusside (10 mM in PBS, pH 7.4) was mixed with 0.1 mL of different concentrations of flexirubin (0.05–10 mM) and Trolox (1 mg/mL) as the standard. The mixture was incubated at 37 °C for 60 min. After incubation, 5 mL of Griess reagent was added, and the mixture was incubated at 25 °C for 30 min in the dark. The absorbance of pink chromophore generated during diazotization of nitric ions with sulphanilamide and subsequent coupling with naphthyl ethylenediamine dihydrochloride was read at 546 nm against the blank. All the tests were performed in triplicates. The percent inhibition activity was calculated using the equation as in Section 2.4.1.

#### 2.4.4. Scavenging Activity of Superoxide Anion Radical (O_2_^−^)

Superoxide radical scavenging activity of flexirubin was measured by reducing nitro blue tetrazolium (NBT) according to the previously reported method with minor modifications [24]. In this assay, the non-enzymatic phenazine methosulfate-nicotinamide adenine dinucleotide (PMS/NADH) system generates superoxide radicals, which reduced NBT to purple formazan. The reaction mixture containing 1 mL of NBT (156 µM), 1 mL of NADH (468 µM) in 100 mM phosphate buffer (pH 7.8) and 0.1 mL of flexirubin in different concentrations were incubated for 15 min at room temperature. The reaction was then initiated by adding 1 mL of PMS solution (60 µM) to the mixture and incubated at 25 °C for 30 min. Trolox was used as the control. The absorbance at 560 nm was measured against blank samples, and the scavenging activity was calculated using the equation as in Section 2.4.1.

#### 2.4.5. Scavenging Activity of Hydroxyl Radical (OH^−^)

Hydroxyl radical scavenging activity of the flexirubin was determined according to the method reported by previous researchers [25]. The reaction mixture that contained 0.1 mL of different concentrations of flexirubin (0,05–10 mM), 1.0 mL of iron-EDTA solution (0.13% ferrous ammonium sulphate 0.26% EDTA), 0.5 mL of 0.018% EDTA, 1.0 mL of DMSO (0.85% in 0.1 mol/L phosphate buffer pH 7.4) and 0.5 mL of 0.22% ascorbic acid was prepared. The tubes were capped tightly and heated in a water bath at 90 °C for 15 min. The reaction was terminated by adding 1.0 mL of ice-cold TCA (17.5%). To the above reaction mixture, 3.0 mL of Nash reagent (75.0 g of ammonium acetate, 3.0 mL of glacial acetic acid and 2.0 mL of acetylacetone were mixed, and distilled water was added to a total volume of 1 L) was added and incubated at room temperature for 15 min for color development. The intensity of the yellow color formed was measured at 412 nm against a reagent blank. Ascorbic acid was used as the standard. The equation determined the percentage of inhibition as in Section 2.4.1.

#### 2.4.6. Lipid Peroxidation Inhibition Activity in a Linoleic Acid System

##### Lipid Peroxidation Inhibition by Ferric Thiocyanate Method (FTC)

The FTC assay was adapted from [26] with minor modifications. A glass-stoppered test tube containing 0.1 mL flexirubin of varying concentrations (0.05–10 mM), 2 mL of 1.5% linoleic acid (in absolute ethanol) and 4 mL of phosphate buffer (0.05 M, pH 7.0) was placed in a water bath at 37 °C in the dark. To 0.1 mL of this reaction mixture at 24 h intervals, 9.7 mL of 75% ethanol and 0.1 mL of 30% ammonium thiocyanate were added. Precisely 3 min after the addition of 0.1 mL of ferrous chloride (0.02 M, in 3.5% HCl) to the reaction mixture, the absorbance was measured at 500 nm. This step was repeated every 24 h until the control reached its maximum absorbance value. The synthetic antioxidant Trolox was used as the standard. The percent inhibition activity was calculated using the equation as in Section 2.4.1.

##### Lipid Peroxidation Inhibition by Thiobarbituric Acid Method (TBA)

The TBA content of the flexirubin was assayed using the method of the previous researchers [26] with modifications. A volume of 0.1 mL of different flexirubin concentrations was mixed with 0.4 mL of linoleic acid (2.5% in absolute ethanol), 0.8 mL of phosphate buffer (0.02 M, pH 7.0) and 0.4 mL distilled water, and kept in the dark at 37 °C for 24 h. A volume of 2 mL of 20% trichloroacetic acid and a volume of 2 mL of 0.67% TBA were added to the incubated flexirubin solution. The mixture was incubated at 37 °C in a water bath for another 24h. Upon incubation, the mixture was centrifuged at 3000 rpm for 20 min. The absorbance of the supernatant was measured at a wavelength of 532 nm. Negative controls without flexirubin and Trolox standards were treated using the same procedure. The inhibition was determined using the equation as in Section 2.4.1.

#### 2.4.7. Ferric Reducing Antioxidant Power (FRAP)

FRAP assay was done according to the method described by the previous researchers [27]. The working solution was prepared freshly before the test by mixing FeCl_3_× 6H_2_O solution (20 mM), acetate buffer (300 mM PH 3.6) and TPTZ solution (10 mM in 40 mM HCl) in the ratio of 2.5: 25: 2.5 (*v/v/v*), respectively, and then warmed at 37 °C. A volume of 0.1 mL of different flexirubin concentrations (0.05–10 mM) was allowed to react with the FRAP solution (2.9 mL) in the dark at room temperature for 30 min. The increase in absorbance of the colored product (ferrous tripyridyltriazine complex) was measured at 593 nm. Working solutions of Trolox were used for calibration. The antioxidant capacity based on reducing ferric ions of the flexirubin was calculated and expressed as µMTrolox equivalents. Ascorbic acid was used as the standard.

#### 2.4.8. Ferrous Chelating Activity

The metal chelating activity of flexirubin was evaluated using the previous researchers’ method [28] with slight modifications. To 0.1 mL of flexirubin (0.05–10 mM), ferrous chloride (0.50 mL, 2 mM) and ferrozine (2 mL, 5 mM) were added. The total volume was adjusted to 3 mL with distilled water, and then the reaction mixtures were shaken vigorously and allowed to stand at room temperature for 10 min. The absorbance of the reaction mixtures was measured at 562 nm. Each flexirubin sample was analyzed in triplicates, and Trolox was used as standard. The ferrous chelating activity was calculated using the equation as in Section 2.4.1.

### 2.5. Molecular Modeling Study

The flexirubin structure was retrieved from the literature [13], and the Trolox structure was extracted from PubChem. Flexirubin and Trolox were prepared by small-molecule tools (ATB web server 3.0) to give the most optimized conformation structures for docking. Cu/Zn-SOD (PDB ID: 1CBJ) was chosen from the RCSB databank for the target protein model. The SOD was prepared by PyMOL macromolecular tools with all water removed and with amino acids completed. SwissDock was used as the docking software to analyze the possible interactions of flexirubin/Trolox with SOD. The binding energy and intermolecular interaction were compared and analyzed using BIOVIA discovery studio visualizer (version 19); https://discover.3ds.com/discovery-studio-visualizer-download.

### 2.6. Statistical Analysis

All the in vitro antioxidant assays were conducted in triplicates. The statistical analysis was performed using IBM SPSS version 25.0. Shapiro–Wilk test was used to determine the normality of data. The independent sample t-test determined the significant differences among the different flexirubin treatments and standard compounds in normally distributed data. For the data that violated the assumption of normality, the comparison using the Mann–Whitney *U* test was used to assess the statistical differences among treatments and standards. A difference was considered statistically significant when *p* ≤ 0.05.

The research was further verified by the evidence that is already present in the relevant field, including:

- A prospective source of antibacterial compounds;
- Bacterial pigments and their applications, Process Biochem;
- Enzymatic activity enhancement of non-covalent modified superoxide dismutase and molecular docking analysis, Molecules;
- In vitro antioxidant activity and antimicrobial activity against biofilm-forming bacteria by the pigment from Desert soil Streptomyces sp D25.

## 3. Results and Discussions

### 3.1. DPPH Radical Scavenging Assay

**Figure 2:**
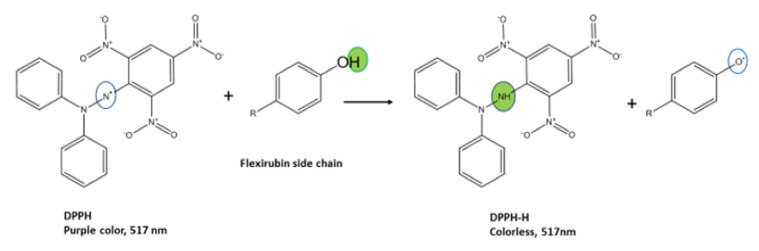
The reaction schemewith flexirubin reduction in the DPPH assay was proposed using Chem Bio Draw Ultra 12.0.

**Figure 3:**
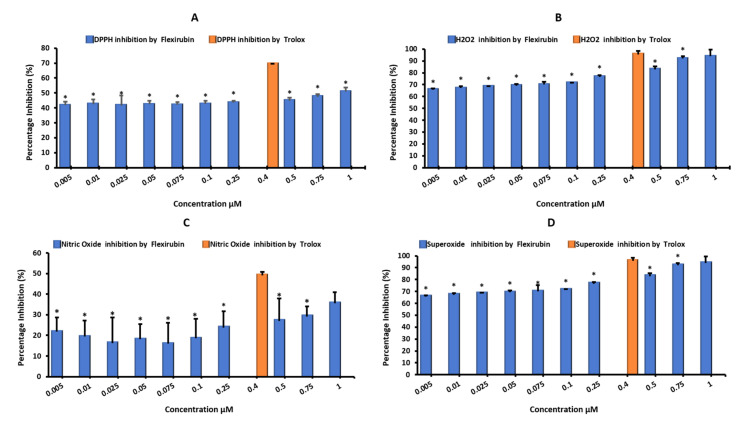

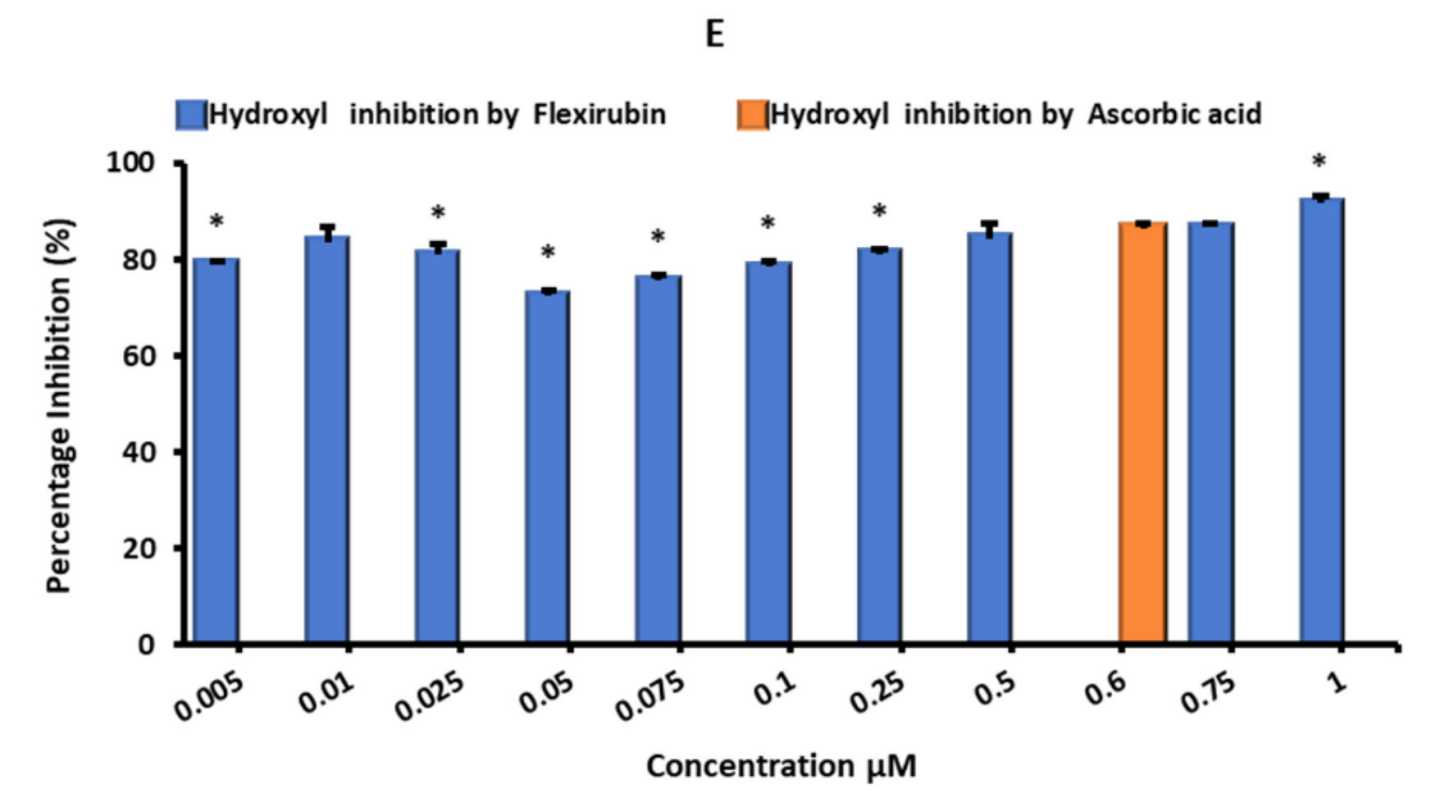
Antioxidant activity of flexirubin extract at different concentrations (0.005–1 μM). (**A**)2-2-Diphenyl-1-picryl-hydrazyl (DPPH) free radical scavenging activity. (**B**) Hydrogen (H_2_O_2_) peroxide scavenging activity. (**C**) Nitric oxide (NO) radical-scavenging ability. (**D**) Superoxide (O_2_^−^) radical scavenging ability. (**E**) Hydroxyl (OH^−^) radical scavenging activity. Each point represents the mean ± SD (*n* = 3); * shows statistically significant differences at *p* ≤ 0.05 in the observed activities when compared with that of Trolox or ascorbic acid standard.

The extracted flexirubin pigment reduced the DPPH radical to the colorless diphenyl picrylhydrazine as shown in Figure 2. It is clear from Figure 2 that the antioxidant activity of the flexirubin can be attributed to the hydrogen atom transfer from the phenol of flexirubin. This observation is in accordance with the results reported in the previous papers [12,29]. Figure 3A–E shows the antioxidant activity of flexirubin at different concentrations (0.005−1 μM)using different in vitro assays. In Figure 3A the highest and lowest DPPH scavenging activities were 70.0 at 0.4 µM and 41.65% at 0.005 µM, respectively. Similar results of DPPH-free radical scavenging potential were obtained from yellow pigment extracted from the bacteria *Arthrobacter gandavensis* [4]. In Figure 3B the highest and lowest scavenging activities were 97.0 at 0.4 µM and 68.0% at 0.005 µM, respectively. In Figure 3C the highest and lowest scavenging activities were 50.0 at 0.4 µM and 17.0% at 0.025 µM, respectively. In Figure 3D the highest and lowest scavenging activities were 98.0 at 0.4 µM and 68.0% at 0.005 µM, respectively. As mentioned, free radicals′ uncontrol production appears to merit most human diseases, including cancer and cardiovascular disease [30]. The good radical scavenging activity of flexirubin was comparable to Trolox (a well-known synthetic antioxidant) and may suggest its use for treating the diseases mentioned above.

### 3.2. Hydrogen Peroxide Scavenging Activity (H_2_O_2_)

In this study, the flexirubin pigment’s ability to scavenge the hydrogen peroxide radicals was determined and compared with Trolox. The higher scavenging activity of 93.85% for flexirubin was observed at 1.0 µM concentration with no significant difference (*p* ≤ 0.05) when compared to the Trolox value of 95.58%. The lower scavenging activity of 65.77% was observed at the tested concentration of 0.005 µM. The H_2_O_2_ scavenging effect of flexirubin increased with the increase in its concentrations (Figure 3B). Hydrogen peroxide, superoxide and hydroxyl radicals detoxification are imperative for protecting biomolecules and synthesizing useful pharmaceutical products [31,32,33] and that role can be played by flexirubin.

### 3.3. Nitric Oxide Radical Scavenging Ability (NO^−^)

**Figure 4:**
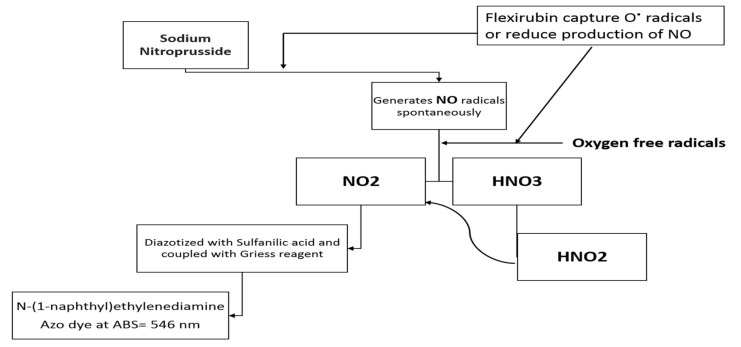
Expected flexirubin mode of action in nitric oxide scavenging assay.

Based on Figure 3C, the evaluation of the NO scavenging activity of flexirubin pigment displayed prominent activity depending on concentration, where the minimum and maximum scavenging activities were 21.74% and 35.46% 0.005 µM and 1.0 µM, respectively. The results displayed significant differences in scavenging percentages (*p* ≤ 0.05) when compared with that of Trolox. In the previous study [34], *Streptomyces* pigment extracted from the *Streptomyces sp.* D25 was another yellow pigment that showed the dose-dependent nitric oxide scavenging activity. Nitric oxide is counting a biological molecule that participates in the regulation of many processes in nervous, cardiac and immunity systems [35,36,37,38]. Bajpai et al. [39] reported that the tested compound must have nitrite scavenging ability to be confirmed as a potential drug. Figure 4 explained the possible mode of action of flexirubin in the NO^−^ assay. Flexirubin may enhance the antioxidant mechanism towards NO free radical scavenging by reducing the production of NO radicals from sodium nitroprusside aqueous solution or capturing oxygen radicals, hence preventing the formation of azo dye that detected at 546 nm.

### 3.4. Superoxide Radical Scavenging Ability(O_2_^−^)

Superoxide anion in the body can lead to another harmful free radical causing cell damage [40]. Superoxide anion interacts with the NO to form the toxic peroxynitrite (ONOO^−^) radical [41]. Superoxide anion can also dissociate from hydrogen peroxide (H_2_O_2_), either suddenly or stimulated by the SOD enzyme [40]. In this study, flexirubin was an effective scavenger of superoxide radical (93.85% at 1.0 µM) in a dose-dependent aspect, while Trolox showed a percentage of 95.58% at a concentration of 0.4 µM. All concentrations of flexirubin were significantly different from Trolox at *p* ≤ 0.05 except for 1.0 µM of flexirubin that showed no significant difference with that of Trolox (Figure 3D). Astaxanthin, a red carotenoid pigment extracted from marine organisms, is well-known to prevent membrane lipid peroxide formation with greater activity than all other known antioxidant compounds [42]. This protective effect is due to its ability to scavenge superoxide anion radicals [43]; fucoxanthin is another familiar marine carotenoid in edible brown sea weeds known for its beneficial biological effects, and its activity to quench superoxide radicals was reported [44]. Such studies suggested that potent superoxide scavenging activity belongs to a specific compound that may lead to many therapeutic benefits.

### 3.5. Hydroxyl Radical Scavenging Activity (OH^−^)

In the presence of metals such as copper or iron ions, hydroxyl radical can be generated from its precursors such as hydrogen peroxide and superoxide anion. Hydroxyl radical can react with aromatic compounds and adds to their double bond to produce a peroxyl radical (by reacting with oxygen) or converting to phenoxy-type radicals (by eliminating water) [45,46]. Hydroxyl radicals can also react in biological systems with various molecules such as lipids, proteins and nucleic acids to cause biomolecules oxidative injuries and cell mutations. Therefore, this makes it a very harmful compound to the cell, leading to many chronic degenerative [47]. Thus, an effective antioxidant is required to compete with hydroxyl radicals and their precursors to either scavenge or block the precursor formation [48]. As revealed in Figure 3E, flexirubin showed hydroxyl radical scavenging activity that depends on its concentration. The capability of the flexirubin to quench hydroxyl radical seems to be again due to the presence of hydrogen donating ability of the flexirubin phenolic ring (Figure 1). The hydroxyl radical scavenging activity of flexirubin showed the maximum inhibition percentage of 91.65 ± 1.38% at 1.0 µM, while ascorbic acid showed 86.42 ± 1.05% inhibition at 0.6 µM. Three concentrations of flexirubin (0.75 µM, 0.5 µM and 0.01 µM) demonstrated no significant difference when compared to ascorbic acid at *p* ≤ 0.05 with inhibition activity of 86.67 ± 0.83%, 84.31 ± 3.07% and 83.58 ± 3.08%, respectively. These results show similarity to the percentage value of hydroxyl radical scavenging capability of Canthaxanthin, the predominant carotenoid pigment produced by *Dietzianatronolimnaea* HS-1 [49].

Briefly, the extracted flexirubin pigment could scavenge free radicals in two manners of actions, i.e., (i) concentration-dependent manner such as scavenging of H_2_O_2_ and O_2_^•^ and (ii) concentration-independent manner such as scavenging of DPPH, NO^•^ and ^•^OH. The best scavenging activity was observed for the highest concentration of flexirubin 1.0 µM in ^•^OH, H_2_O_2_ and O_2_^•−^ assays. In the second stage, flexirubin has good DPPH scavenging ability at different concentrations of flexirubin. Finally, the evaluation of NO scavenging activity of flexirubin pigment showed prominent activity, and the results showed significant differences in scavenging percentages compared with that of Trolox. There are around 19 in vitro antioxidant assays used to estimate the unknown compounds′ antioxidant activity. The authors used five assays of scavenging in the current study depending on the radical type, general free (DPPH) and specific radicals’ hydroxyl, hydrogen peroxide, nitric oxide and superoxide; these radicals can be available in any living cell. Moreover, no redox reaction before on flexirubin-type pigment makes this manuscript a good review for scientists interested in working with this type of pigment. The significance of each redox reaction already clears in the current manuscript discussion, and the literature is full of papers that used all these redox reactions integrated to give a comprehensive picture of their compound’s activity.

### 3.6. Inhibition of Lipid Peroxidation

**Figure 5:**
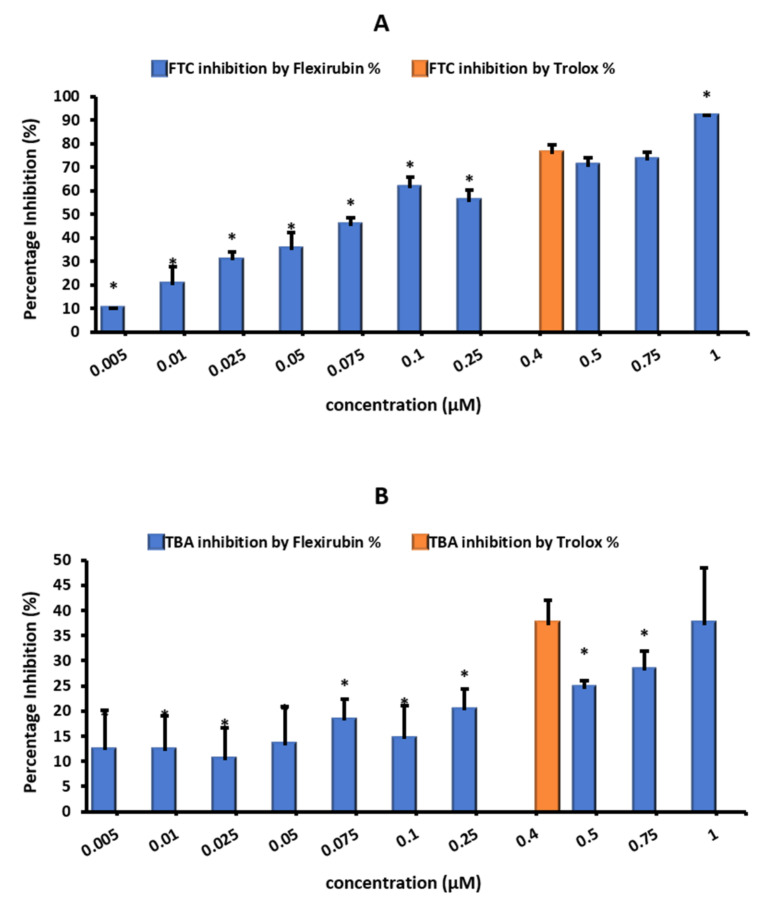
Inhibition of lipid peroxidation by flexirubin extract at different concentrations (0.005–1 μM) using two methods. (**A**) ferric thiocyanate (FTC) method. (**B**) Thiobarbituric acid (TBA) method. Each point represents the mean ± SD (*n* = 3); * shows statistically significant differences at *p* ≤ 0.05 in the observed activities when compared with that of Trolox standard.

Another important category of in vitro assays used is the evaluation of lipid peroxide inhibition. Lipid peroxidation is an upshot of oxidative degradation of cell membrane lipids. This process consists of a series of free radical-mediated chain reactions and leads to the pathogenesis of major degenerative diseases and several damages [50]. In this study, both FTC and TBA methods were used to measure the peroxidation inhibiting activities of flexirubin against linoleic acid’s system. At the early stage of lipid oxidation, the FTC method defines the amount of peroxide produced, whereby a higher antioxidant activity appears as lower absorbance. In this reaction, the linoleic acid oxidation produces peroxides that interact with Fe^2+^ to form Fe^3+^. Fe^3+^ ions react with SCN- to give a thiocyanate complex that shows the maximum absorbance at 500 nm. Figure 5A shows the changes in the activity for each flexirubin concentration that reached its maximum absorbance after 48 h, in comparison with the Trolox. Flexirubin at 1.0 µM had the highest significant inhibition activity (91.03 ± 1.00, *p* ≤ 0.05) to indicate the lowest peroxide concentration and the highest antioxidant activity level. At 0.75 µM and 0.5 µM flexirubin concentrations, significant differences did not prevail compared with Trolox’s (*p* ≤ 0.05).

Peroxides are gradually decomposed to malondialdehyde (MDA) during the oxidation process, a useful biomarker in the latter stage of lipid peroxidation measured by the TBA method [26]. Figure 5B showed that the antioxidant activity of flexirubin being concentration-dependent. Antioxidant activity at a high concentration of flexirubin (1.0 µM) showed no significant difference (*p* > 0.05), while the rest of the flexirubin concentrations were significantly lower (*p* ≤ 0.05) than that of Trolox activity. The results of the lipid peroxidation assay suggested that flexirubin has potent antioxidative effects in preventing lipid peroxidation. The antioxidant activities of current flexirubin are similar to flexirubin-type pigment isolated previously from *Fontibacter flavus* YUAB-SR-25 [51].

### 3.7. Ferric Reducing Antioxidant Power (FRAP)

**Figure 6:**
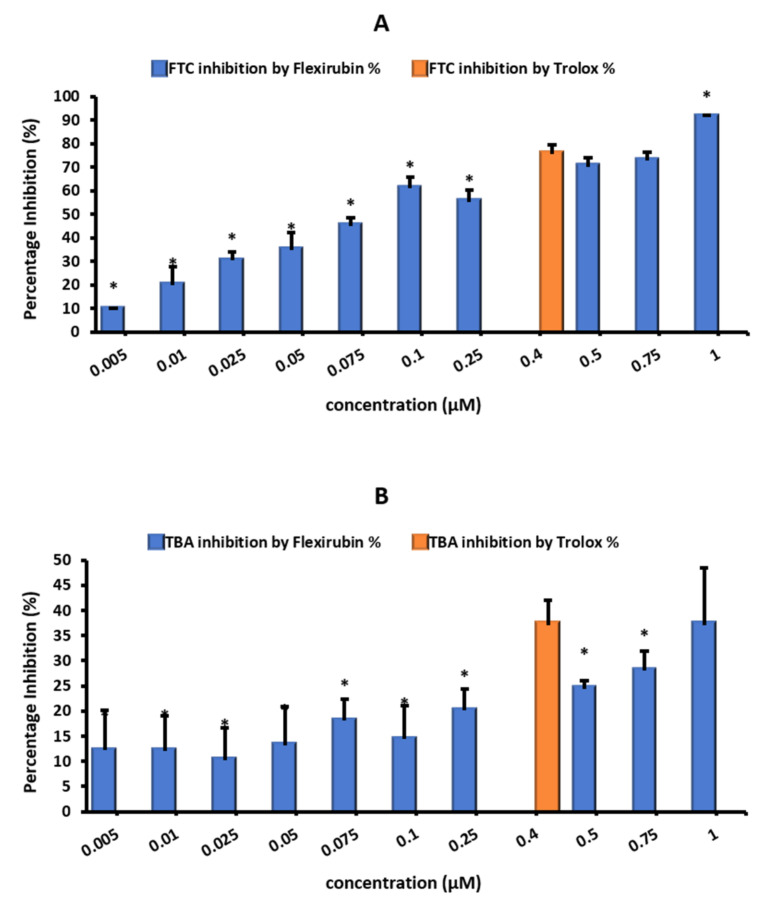
Antioxidant activity of flexirubin extracts at different concentrations (0.005–1 μM) using a metal complexation and reducing abilities. (**A**) Ferric reducing antioxidant power (FRAP). (**B**) Ferrous complexation ability. Each point represents the mean ± SD (*n* = 3); * shows statistically significant differences at *p* ≤ 0.05 in the observed activities when compared with that of Trolox or Ascorbic acid standard.

The metal-reducing capacity serves as a significant potential antioxidant activity indicator of any tested compound [52]. In FRAP, the tested compound reacts with a ferric tripyridyltriazine (Fe^3+^-TPTZ) complex generates a blue ferrous tripyridyltriazine (Fe^2+^-TPTZ) complex, which can be measured spectrophotometrically at 593 nm. Generally, reducing property is associated with compounds that exert their action by donating a hydrogen atom and breaking the free radical chain. In this assay, the results are expressed as Trolox equivalents (TE) in µM using ascorbic acid as a standard compound. None of the different flexirubin concentrations showed greater activity than ascorbic acid, and flexirubin had a very low ability to reduce Fe^3+^ to Fe^2+^^,^ which can be inferred from significant differences (*p* ≤ 0.05) in their FRAP median values among different concentrations tested as compared to the standard (Figure 6A). Flexirubin observed a range of reduction abilities similar to Indian medicinal plants mentioned by previous researchers [53].

### 3.8. Ferrous Complexation Ability

In vivo, transition metals such as iron play an important role in the generation of ROS. Fe^2+^ ions can induce lipid peroxidation by catalyzing the Haber–Weiss reaction. Fe^2+^ is oxidized to Fe^3+^ by oxygen molecule (O_2_) to produce peroxide, which reacts with a hydrogen ion (H^+^) to produce H_2_O_2_. Then H_2_O_2_ reacts with Fe^2+^, and ^•^OH is generated via Fenton reaction. Again ^•^OH is produced by hydrogen peroxide in the presence of Fe^3+^/Fe^2+^ (Haber–Weiss reaction) [54]. Hence, cations that can make complexes with ironact as a secondary antioxidant by preventing iron as a catalyst for the redox process [28]. The complexation ability of a tested compound is an important index of its antioxidant activity. As shown in Figure 6B, flexirubin has a complexation activity that ranged from 30.02–33.23% for 1.0 µM and 0.075 µM, respectively. The ferrous complexation activity of Trolox was equal to 33.61 ± 1.11; this value showed a significant difference at (*p* ≤ 0.05) compared to three higher concentrations of flexirubin, i.e., 1.0, 0.75 and 0.5 µM, while showing no significant difference compared to other concentrations of flexirubin (*p* > 0.05).

The presence of a phenolic side chain or double bond conjugation system in flexirubin structure might be the reason for achieving such a metal complexation activity. A previous study reported that chestnut shell melanin fractions’ peroxidation inhibition activity might be mediated by their iron-binding capacity [55]. Accordingly, results suggested that flexirubin peroxidation inhibitory ability may be associated with its iron-binding capacity [56]. Briefly, flexirubin showed concentration-dependent inhibition of lipid peroxidation through FTC and TBA assays. The highest concentration of flexirubin showed higher significant inhibition of FTC as compared to Trolox. Moreover, flexirubin has a good ferrous-complexation ability and reversely low ferric reducing activity.

### 3.9. Flexirubin Computational Modeling Studies

**Figure 7:**
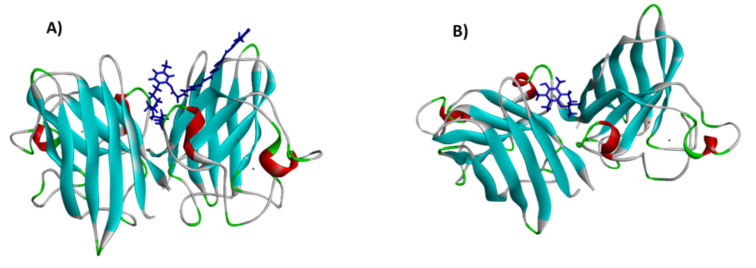
Three-dimensional structure of (**A**) Flexirubin and (**B**) Trolox to cavity site of Cu/Zn superoxide dismutase enzyme generated by BIOVIA discovery studio visualizer (version 19).

From in vitro assays, the proposition that flexirubin has antioxidant activity via scavenging free radicals appears supported. Therefore, it seems reasonable to estimate the mode of interactions between flexirubin and biomolecules at the molecular level and first insight into the possible mechanism of flexirubin as an antioxidant. Moreover, it is necessary to compare the docking results with well-known antioxidant compounds [57]. Trolox was chosen as it is a well-known standard used for all in vitro assays in this study. Docking using SwissDock was performed to investigate the possible interaction mode and determine the most stable complex between SOD and chosen ligands (Figure 7).

The binding energy (ΔG) of flexirubin was −9.99, while theΔG of Trolox was −7.20, indicating the more stable binding energy of flexirubin with SOD enzyme Trolox.

**Figure 8:**
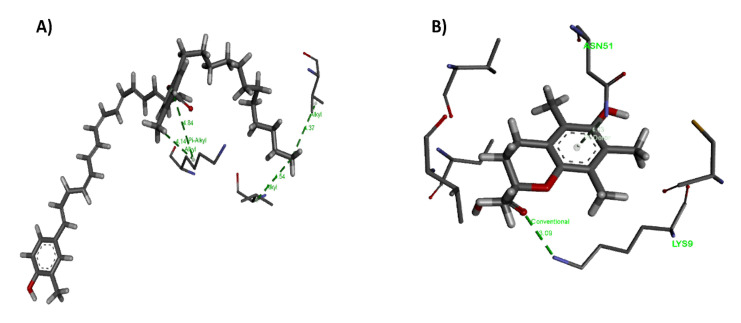
Different bond distances of (**A**) Flexirubin and (**B**) Trolox with Cu/Zn superoxide dismutase enzyme generated by BIOVIA discovery studio visualizer (version 19).

**Figure 9:**
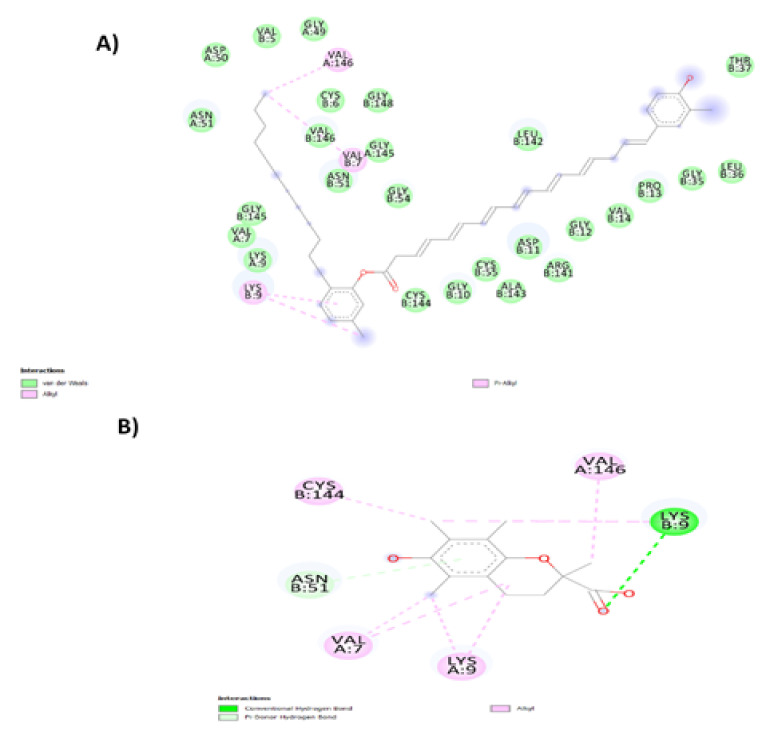
(**A**) Flexirubin and (**B**) Trolox interaction with amino acids on Cu/Zn superoxide dismutase enzyme chains generated by BIOVIA discovery studio visualizer (version 19).

The negative values of ΔG revealed that the forces between Flexirubin/Trolox with SOD are primarily spontaneously formed hydrogen bonding and van der Waal’s forces. Flexirubin/Trolox posed in the binding pocket cavity where the protein is located, while the enzyme metal active sites remained unaffected. Hence, the activity of the SOD enzyme would not change or reduce due to the complex formation with flexirubin or Trolox. Based on Figure 4, both compounds bound into the Cu/Zn-SOD interface and interacted with two subdomains. For the flexirubin-SOD complex (Figure 8A and Figure 9A), the interaction occurred via two alkyl bonds on Val146. A and Val7.B with the bond distance of 4.37 Å and 4.54 Å, respectively, and one bialkyl non-covalent interaction on Lys9.B with the bond distance of4.14 Å and 4.84 Å.

The majority of interactions were van der Waals forces with amino acids on both A and B subdomains. For Trolox-SOD (Figure 8B and Figure 9B) complex, the interaction occurred via two alkyl bonds with Val146. A and Cys144.B and two hydrogen bonds with Lys9.A and Val7. A. Comparing the G-score of the flexirubin to the in vitro data concluded that the docking could not predict the antioxidant activity under the experimental methods reported in this study; however, it gives the first-ever insight into the interactions between the flexirubin and the active site of SOD enzyme [58] and clarifies the flexirubin and Trolox behaviors as antioxidants.

## 4. Conclusions

In the present study, various analytical methods are combined to develop a broad picture of the flexirubin antioxidant activity status. Different assays should be integrated into a panel and used for antioxidant activity evaluation; the real fact supports this recommendation that the result of antioxidant activity could be noticeably different from one assay to another. The antioxidant potential via hydrogen donating ability of flexirubin has been proven through the assessment using different assays such as radical scavenging activities, lipid peroxide inhibition and ferrous chelating ability. Moreover, the docking studies indicate that the binding of flexirubin into the cavity of the SOD enzyme does not affect its activity. These results confirm the efficacy of flexirubin as a significant source of natural antioxidants, which might help prevent the progress of various oxidative stress-induced diseases. However, the in vivo antioxidant activity needs to be investigated thoroughly before its practical application. To the best of our knowledge, this study is the first on free radical scavenging activity, antioxidant properties and computational study of flexirubin from *C. artocarpi*.

## Data Availability

No data is available except included in this article.

## Sample Availability

Samples of the compounds by Wan Azlina Ahmad are available.
